# The value of leukapheresis for treatment of priapism as presenting feature of chronic myeloid leukemia—Case report and review of literature

**DOI:** 10.1002/jha2.545

**Published:** 2022-08-27

**Authors:** Marleen G. A. M. van der Velde, Sanne M. B. Tiellemans, Heleen de Lil, Laurens Nieuwenhuizen

**Affiliations:** ^1^ Department of Internal Medicine Máxima MC Veldhoven/Eindhoven The Netherlands; ^2^ Department of Health Services Research CAPHRI School for Public Health and Primary Care Aging and Long Term Care, Maastricht Limburg The Netherlands; ^3^ Department of General Practitioning RadboudUMC Nijmegen The Netherlands

**Keywords:** chronic myeloid leukemia, hyperleukocytosis, leukapheresis, leukostasis, priapism

## Abstract

Priapism is a rare presenting feature of chronic myeloid leukemia (CML) in male patients. Treatment aims to relieve symptoms and to prevent erectile dysfunction. Several treatment modalities exist, however no standard treatment is recommended. We evaluated literature concerning different treatment approaches and evaluate the value of leukapheresis in treatment of priapism. The literature search resulted in 57 included articles, consisting of 53 studied patients. Patients had a mean age of 25.3 years, average time from onset to presentation at the hospital was 2 days, and mean white blood cell (WBC) count was 344 × 10^9^/L. Most patients (67.9%) were treated with a combined approach (different modalities were radiological, urological, and oncological treatment). Twelve patients, with a mean WBC count of 365 × 10^9^/L, received leukapheresis. Only two of them reported erectile dysfunction after treatment. Priapism is an urological emergency requiring urgent multidisciplinary treatment. We highlight the importance of local urological therapy combined with systemic therapy for CML. Therapeutic leukapheresis should be applied when available and with no other contraindications.

## 1 INTRODUCTION

Priapism as presenting feature of chronic myeloid leukemia (CML) is rare, approximately 1%–2% in all male cases of CML [1]. Priapism is an urological emergency characterized by full or partial penile erection, lasting more than 4 h and unrelated to sexual stimulation.

Two main types of priapism can be distinguished: ischemic (low flow) and non‐ischemic (high flow) priapism, both with their own appropriate management. Ischemic priapism is a type of compartment syndrome with reduced intracavernous blood flow, leading to stasis, acidosis, and hypoxia [1]. If untreated, it can lead to irreversible damage and fibrosis, resulting in erectile dysfunction (ED). Non‐ischemic priapism is due to unregulated cavernous flow, most commonly caused by trauma to penis or perineum. This results in increased arterial flow that overwhelms venous outflow, leading to stasis of oxygenated blood. This makes irreversible damage or fibrosis rare [1].

In ischemic priapism cases, most are idiopathic; however, 20% is caused by diseases such as sickle cell disease, CML, acute myeloid leukemia (AML), and polycythemia vera [2].

Ischemic priapism results from sludging of blood within the corpora, which results in ischemia and smooth muscle hypoxia leading to pain. In CML patients, hyperleukocytosis is considered to be the cause of sludging, especially when the white blood cell (WBC) count exceeds 200 × 10^9^/L [1]. First, high leukocyte levels and aggregation in corpora cavernosa lead to vascular obstruction of the dorsal penile veins, which result in vascular stasis and reduced venous outflow. Venous congestion in the corpora is also a result of compressed intra‐abdominal veins due to splenomegaly. Additionally, infiltration of the sacral nerves and central nerve system with leukemic cells also contribute to priapism in CML [3].

To our knowledge, there is no standard treatment recommended for leukemic priapism and evidence for different approaches derived from case reports and small case series. We describe a case of a young male presenting with priapism as a presenting feature of CML. Furthermore, we have examined the evidence for different treatment modalities and evaluate the value of leukapheresis in management of leukemic priapism.

## 2 CASE DEFINITION

A 27‐year‐old previously healthy male presented to the emergency department with painful priapism lasting 6 h at time of presentation. He stated that during the past 4 months, he had been experiencing numerous involuntary, prolonged painful erections, without sexual stimulus. There was no history of trauma, medication use, fever, or weight loss. The patient reported progressive fatigue over the past few months. Upon presentation, the patient had a fever of 38.6°C and tachycardia of 130 bpm with normal blood pressure. The physical examination revealed a palpable spleen over 10 cm below the left costal margin, while lymphadenopathy or hepatomegaly was not present. The penis was erect and tender with venous engorgement. Laboratory findings showed hemoglobin (Hb) of 4.1 mmol/L (6.6 g/dl), WBC count of 502 × 10^9^/L, and platelet count of 164 × 10^9^/L. A peripheral blood smear showed immature leukocytes in various stages of differentiation (blast cells 7%, promyelocytes 9%, myelocytes 18%, metamyelocytes 3%, and neutrophils 51%), no platelet clumps were seen. Bone marrow aspiration showed hyperplasia of the myeloid cells without blast increase, correlating to chronic phase of CML. The SOKAL‐score was 1.26, indicating high risk [4]. Initial management of the priapism done by cavernosal aspiration achieved full detumescence. At the same time, the patient started with leukapheresis and hydroxycarbamide 3 g daily. Hyperhydration was started with rasburicase to prevent tumor‐lysis syndrome. Fever was treated with intravenously antibiotics, second‐generation cephalosporins. A total of three leukapheresis sessions were needed. Detection of a breakpoint cluster region‐Abelson (BCR‐ABL) major fusion transcript confirmed the diagnosis of CML presenting with priapism, and treatment with Imatinib was started. There was no recurrence of priapism. He tolerated treatment well and without further complications, he was subsequently discharged home with a WBC count of 79 × 10^9^/L. Patient was a poor responder to Imatinib and was subsequently switched to Dasatinib. After start of Dasatinib, patient complained of brain fog and concentration problems and therefore switched to Bosutinib. At 1‐year of follow‐up, BCR‐ABL was decreased below 0.1% and Bosutinib was continued. Patient reported no complaints of sexual dysfunction.

## 3 METHOD

### 3.1 Study design

We analyzed literature describing treatment of priapism in CML. The search was restricted to literature including any treatment of leukemic priapism in patients with CML. Only articles written in English or Dutch with full‐text availability were included. In addition, bibliographies of relevant systematic reviews or overview articles identified during the search were also reviewed to identify additional relevant studies.

### 3.2 Information sources and search

The PubMed (Medline Ovid) database was searched, and the latest comprehensive search was conducted on January 5, 2022.

The search terms used for searching the database were “Chronic Myeloid Leukemia,” “Chronic myelocytic leukemia,” “BCR ABL positive,” and “Priapism.” The search strategy and queries were assessed by a biomedical information specialist for the PubMed database (see Table S1, for the complete syntaxes).

### 3.3 Study selection

Two researchers (Marleen van der Velde and Sanne Tiellemans) independently screened all identified studies for inclusion based on title and abstract, by using Rayyan QCRI [5]. Of the selected studies, the full text was assessed by the same investigators. Any conflicting results in the selection process were discussed until consensus was reached. We decided to only include studies performed after January 1, 2000, because with the advent of the tyrosin kinase inhibitors (TKIs), the treatment of CML changed drastically. All studies which included any sort of treatment of priapism in CML were included.

### 3.4 Data collection

From each included article we extracted, if available, the following data: authors, year, and journal of publication. When the article was a case report, we included the following patient characteristics: age, time to first presentation, WBC count, time to treatment, known history of CML, treatment strategy including the use of leukapheresis, resolution, and ED. We analyzed these data using descriptive statistics of the observed data. Continuous data are presented as mean and standard deviation (SD) or median and first–third quartile, and nominal data are presented as number and percentage. All data were analyzed using the software Statistical Package for the Social Sciences, version 25 (IBM Corp., NY, USA). For articles consisting of patients cohort or systematic reviews, a short summary of the objective and results was made. Data were extracted by two researchers (Sanne Tiellemans and Marleen van der Velde).

### 3.5 Data analysis

The primary objective was to provide an overview of different approaches in treating priapism caused by hyperviscosity and evaluate the value of leukapheresis as treatment modality. Treatments were categorized based on oncological or urological approach, and whether there was a stepped‐up approach. We also reported if resolution was reached and whether ED was present after treatment.

## 4 RESULTS

### 4.1 Different approaches in management of ischemic priapism

Treatment modalities in low flow priapism due to hyperviscosity consist of radiological, urological, and oncological management. Radiological management of priapism consists of localized radiation therapy, most often in fractionated doses to the penis to resolve priapism. Irradiation to the spleen to reduce the venous outflow obstruction caused by splenomegaly is another option in treating priapism caused by CML [6]. Possible complications of penile irradiation consists of urethra fibrosis causing lower urinary tract symptoms and ED due to reduced penile blood flow. In management of priapism caused by CML, few case reports mention using irradiation as treatment modality. Hematological disorders tend to be more chemo‐ and radiation sensitive; however, there is no in‐depth literature available evaluating the optimal dose and duration or value of radiation therapy in malignant priapism [7].

The two main treatment strategies are therefore urological and oncological treatment. The main goal of treatment is to pursue detumescence as quickly as possible, to prevent irreversible damage and fibrosis leading to problems with erectile function or future episodes of persistent and prolonged priapism (stuttering priapism). The modalities of urological management consist of aspiration, intracavernous sympathomimetics, shunts, and penile prothesis [1, 8]. Initial urological management may include aspiration (with or without irrigation) or intracavernous injection of sympathomimetics. Unilateral aspiration is sufficient as both corpora are interconnected, and it has a success rate of approximately 30% [1]. Evacuation of blood by corporal aspiration relieves the compartment syndrome of the penis [9]. If priapism persists after aspiration, intracavernous injection with phenylephrine or epinephrine can be performed combined with irrigation, this has a success rate of 43%–81%. A higher resolution rate is achieved after injection of a sympathomimetic agent, and this lowers the risk of post‐priapism ED [9]. Only when these two techniques fail, surgical shunts should be considered. Extended durations of ischemic priapism are less likely to resolve with solely injection/irrigation therapy alone, in which immediate surgical shunting must be considered. The goal of these surgical procedures are to create a channel allowing the deoxygenated blood to drain from the corpora cavernosa. Four types of shunts are used in daily practice: percutaneous distal, open distal, open proximal, and vein anastomoses shunts. First choice of shunt procedure is the percutaneous distal shunt (Winter's/corporoglanular shunt), wherein a large needle or scalpel is inserted percutaneously through the glans. The shunts will close with time, but it may lead to ED.

Oncological management includes cytoreductive therapy with high‐dose hydroxycarbamide and when BCR‐ABL positive followed by the induction of TKIs. Initial management is sometimes combined with leukapheresis to reduce hyperviscosity, but its popularity has decreased with the introduction of TKIs [7, 10]. Leukapheresis is a blood filtrating process that separates and removes WBC from the circulated blood, which reduces the WBC count up to 60% [11]. Due to the lowering of WBC, this procedure reduces the degree of tissue infiltration and hyperviscosity caused by hyperleukocytosis and leukostasis, relieving acute symptoms such as priapism. It should be considered especially in the initial phases of treatment, where the maximum effect of cytoreductive therapies is yet to come [6]. However, leukapheresis is not available in every setting and costs are high. Most commonly, these therapies are combined with supportive treatment with hyperhydration and allopurinol to prevent tumor‐lysis syndrome. Although only 35% of cases managed with systemic treatment alone resulted in ED, it is strongly recommended that systemic treatment of the underlying disorder should not be undertaken as single treatment for low flow priapism. Ischemic priapism is a compartment syndrome and requires direct local treatment of the penis [8].

A two‐phase algorithm is proposed by Chisick et al. [11] in order to standardize treatment in cases where priapism as presenting feature of CML is suspected. A multidisciplinary assessment is recommended with simultaneous initiation of hyperhydration, allopurinol, and cytoreductive management, next to specific ischemic priapism treatment consisting of prophylactic anticoagulants, sympathomimetic agents, and leukapheresis.

### 4.2 Study selection

**FIGURE 1:**
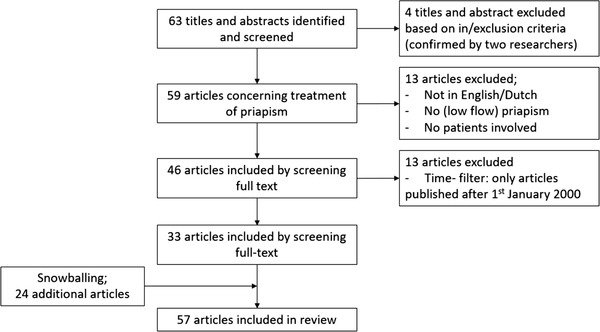
Flow diagram for the included articles

The initial search provided 63 articles with no duplicates. The screening of title and abstracts led to the selection of 59 articles concerning treatment of priapism. Following further screening of these articles, 13 articles were excluded because these were not in English or Dutch, the article did not concern (low flow) priapism, did not include patients or the full text was not available for the researchers. After applying the time filter, 33 articles were included in this review of literature. Of all 63 articles, the researchers analyzed the reference list to identify additional papers which met the inclusion criteria, 24 additional articles concerning treatment of malignant priapism were included. We evaluated a total of 57 articles, 48 case reports consisting of 53 patients, five retrospective studies, two prospective observational studies, and two reviews (Figure 1). For an overview of included articles, see Tables 1 and 2.

**TABLE 1:** Overview of included case reports per patient

First author	Year of publication	Age	First presentation	WBC (×10^9^/L)	Time to treatment	CML history	Use of leukapheresis	Radiation therapy	Management	Resolution	ED
Only oncological treatment											
Atas [12]	2018	18	Y	215	5 days	N	Y	N	Oncologic: hyperhydration, allopurinol, later leukapheresis	Y, after 5 days	NS
Castagnetti [13]	2008	9	Y	509	Several days	N	Y	N	Oncologic: cytoreduction therapy, antibiotics, anticoagulants, leukapheresis	Y	NS
		9	Y	169	NS	N	N	N	NB: stuttering priapism. Oncologic: cytoreduction therapy, anticoagulant	Y	NS
		9	Y	472	9 h	N	Y	N	Oncologic: cytoreduction therapy, antibiotics, anticoagulants, leukapheresis	Y	NS
Ergenc [14]	2015	18	Y	100	72 h	N	Y	N	Oncologic: imatinib, allopurinol, hyperhydration, leukapheresis	Y	Y
Kumar [15]	2017	19	Y	240	21 days	N	N	Y	Oncologic: hydroxycarbamide, busulphan. Radiation: penile radiation	Y	NS
		38	Y	397	24 h	N	N	N	Oncologic: hyperhydration, busulphan	Y	N
Musa [16]	2017	18	Y	199	12 days	N	N	N	Oncologic: hydroxycarbamide, allopurinol	Y, after 4 weeks	Y
Ocheni [17]	2010	30	Y	356	4 days	N	N	N	Oncologic: cyclophosphamide and hydroxycarbamide	Y, not stated when	NS
Veljkovic [18]	2012	16	Y	320	1 day	N	Y	N	Oncologic: anticoagulant, cytoreductive chemotherapy. After no effect leukapheresis, immediate improvement. Imatinib	Y, after 13 days	NS
First oncological, later urological treatment											
Becerra‐Pedraza [19]	2018	52	Y	282	6 days	N	N	N	Urologic: aspiration, irrigation. Surgery shunt. Oncologic: hyperhydration and allopurinol	NS	NS
Gupta [20]	2009	12	Y	364	48 h	N	N	N	Oncological: hyperhydration, hydroxycarbamide. Urological: injection terbutaline	Y, 1 day	NS
Morano [21]	2000	23	Y	660	3 days	N	Y	N	Oncologic: hydroxycarbamide, leukapheresis. Urologic (started after 3 days): aspiration. After conspicuous edema → shunt surgery	Y, slight clinical improvement after puncture	Y
Combined treatment/unknown timeline											
Gaye [22]	2020	46	Y	526	48 h	N	N	N	Urologic: puncture, injection phenylephrine. Oncologic: hydration, allopurinol, hydroxycarbamide, imatinib	NS	NS
Hazra [23]	2013	14	Y	227	24 h	N	N	N	Urologic: aspiration, irrigation and phenylephrine injection. Oncologic: hyperhydration	Y, after 5 days	NS
Huei [24]	2018	28	Y	294	2 days	N	N	N	Urologic: aspiration, irrigation, injection with phenylephrine. Corporaglandula shunting. Oncologic: hyperhydration, cytarabine, hydroxycarbamide	After aspiration short detumescence, but reoccurred hours later. Following drainage: flaccid	NS
Mishra [25]	2020	24	Y	207	5 days	N	Y	N	Oncologic: hydroxycarbamide, hydration and daily leukapheresis (five sessions). Urologic: aspiration (three times), distal shunt	Y, after 7 days	Y
Purohit [26]	2021	17	Y	386	2 days	N	N	N	Urologic: aspiration and irrigation. Oncologic: (after complete detumescence) hydroxycarbamide and imatinib	Y	N
Sossa Melo [27]	2021	47	Y	222	8 h	N	N	N	Urologic: injection, aspiration and irrigation. Distal surgical shunt. Oncologic: hydration, hydroxycarbamide and allopurinol. Dasatinib	Y	NS
Syarif [28]	2021	27	Y	620	10 days	N	N	N	Urologic: winter procedure (shunt), aspiration and injection. Oncologic: hydroxycarbamide and allopurinol	Y	Y
First urological, later oncological treatment											
Abd El Salam [29]	2019	30	Y, just dis‐continued treatment CML	210	16 h	Y	N	N	Urologic: aspiration and irrigation with ephedrine. Oncologic: hydroxycarbamide, hydration and allopurinol	Y, after aspiration	Y
Avci [30]	2005	55	Y	184	8 h	N	N	N	Urologic: aspiration and ephinephrine irrigation, shunt procedure. Oncologic: hydroxycarbamide, allopurinol and hydration	Y, after shunt	NS
Chang [31]	2003	21	Y	217	NS	N	N	N	Urologic: aspiration, irrigation, injection with epinephrine. Oncologic: hydroxycarbamide, interferon α‐2a. Allopurinol. Hyperhydration	Y	N
Clark [32]	2018	13	Y	350	3 days	N	Y	N	Urologic: injection with phenylephrine, three corporal irrigation, shunt surgery. Oncologic: leukapheresis, hyperhydration, hydroxycarbamide, allopurinol	N, still phallus rigidity and tenderness	NS
Dhanju [14]	2019	18	Y	363	14 h	N	N	N	Urologic: aspiration and irrigation with phenylephrine. Oncologic: hydroxycarbamide, allopurinol and later imatinib	Y, after aspiration	N
Dhar [33]	2019	52	Y	239	4 h	N	N	N	Urologic: needle aspiration, shunt surgery. Oncologic: hydroxycarbamide, imatinib, allopurinol, hyperhydration	Y	N
Dogra [34]	2004	18	Y	320	10 days	N	N	N	Oncologic: hyperhydration, allopurinol, 6‐mercapropurine. Urologic: shunt surgery	Partially	Y
Ervie [35]	2015	22	Y	185	9 h (stuttering priapism existed for a month prior to visitation)	N	N	N	Urologic: aspiration and terbutaline injection. Oncologic: hydroxycarbamide, allopurinol and prednisone, later imatinib	Y	NS
Farhan [36]	2015	35	Y	378	30 h	N	N	N	Urologic: aspiration and irrigation. Oncologic: hydration, allopurinol and hydroxycarbamide and later imatinib	Y, after 5 days	NS
Gupta and Agrawal [37]	2008	55	Y	420	2 days	N	N	N	Urologic: shunt surgery. Oncologic: hydroxycarbamide	NS	NS
Jameel [38]	2009	21	Y	316	8 h	N	N	N	Urologic: cavernosa aspiration, irrigation, injection with epinephrine. Oncologic: hydroxycarbamide, allopurinol, hyperhydration	Y	N
		55	Y	282	12 h	N	N	N	Urologic: cavernosa aspiration. Oncologic: hydroxycarbamide, allopurinol, hyperhydration	Y	NS
Khan [39]	2018	16	Y	615	11 days	N	N	N	Oncologic: hydroxycarbamide, aspirin, allopurinol, hyperhydration. Urologic: aspiration and washes	Y	N
Manuel [40]	2007	20	Y	708	Developed during hospital stay	N	Y	Y	Urologic: aspiration and injection with phenylephrine. Oncologic: leukapheresis and oral hydroxycarbamide. Induction chemotherapy. Radiation: local	Y	NS
Minckler [41]	2017	18	N^a^	588	6 h	N	N	N	Urologic: aspiration and irrigation. Oncologic: hydroxycarbamide	Y, after aspiration	NS
Narendra [42]	2011	11	Y	290	12 h	N	N	N	Urologic: conservative management was advised due to short duration, mild pain, and lack of ischemia. Oncologic: hydration, allopurinol, and hydroxycarbamide. Later imatinib	NS	N
Nerli [43]	2016	19	Y	297	24 h	N	N	N	Urologic: aspiration, irrigation, injection with phenylephrine. Oncologic: hydroxycarbamide, imatinib, allopurinol	Y	NS
Rajabto [44]	2020	44	Yes	399	4 days	N	N	N	Urologic: puncture, injection ephinephrine. Oncologic: hydration, allopurinol, hydroxycarbamide, imatinib	Y	Y
Patil [45]	2016	22	Y	157	8 h	N	N	N	Urologic: ultrasound‐guided cavernosa aspiration. Oncologic: imatinib, hyperhydration	Y, after 1 month	NS
Ponniah [46]	2004	19	Y	294	18 h	N	Y	N	Urologic: aspiration. Oncologic: cytotoxic medication, leukapheresis	Y	N
Qu [47]	2019	18	Y	257	72 h	N	N	N	Urologic: aspiration, injection with phenylephrine. Shunt surgery. Oncologic: imatinib, hyperhydration	Y, after surgery	N
Sachdeva [48]	2020	14	No, recurrent in 2 months	458	6 days	N	N	N	Urologic: aspiration and irrigation, later bilateral cavernotomy. Oncologic: hydration, allopurinol, hydroxycarbamide, imatinib	Y	Y
Shaeer [3]	2015	21	Y	410	6 days	N	Y	N	Urologic: aspiration, irrigation, sildenafil citrate. Oncologic: imatinib, leukapheresis,	NS	N
Swapna [49]	2017	18	Y	144	4 days	N	Y	Y	Urologic: aspiration, irrigation, injection with phenylephrine. Radiation: local. Oncologic: leukapheresis, hydroxycarbamide, allopurinol. Initiation of imatinib	Y, after leukapheresis	NS
Thakur [50]	2019	15	Y	135	2 days	N	N	N	Urologic: aspiration, irrigation, phenylephrine injection, distal shunt procedure, later proximal corporospongiosal shunt. Oncologic: hydration, allopurinol, imatinib	Y, after two days	NS
Ullah [51]	2018	22	Y	219	4 days	N	N	N	Urologic: aspiration and irrigation. Oncologic: hydroxycarbamide, interferon α‐2a and allopurinol with hydration	Y, after urologic treatment	NS
Only urological treatment											
Almaeena [52]	2016	36	Y	231	NS	N	N	N	Urologic: aspiration^b^	Y	NS
Ammouri [53]	2019	25	Y	501	16 h	N	N	N	Urologic: aspiration	Y	NS
Htun [54]	2008	21	Y	619	72 h	N	N	N	Urologic: aspiration, shunt surgery	Y, partial tumescence	NS
Ocheni [17]	2010	36	Y	456	34 h	N	N	N	Urologic: aspiration	Y	NS
Sareen [55]	2018	17	Y	377	Recent onset	N	N	N	Urologic: injection of phenylephrine, aspiration	Y	NS
Sun [56]	2019	27	N^c^	450	9 h	Y^d^	N	N	Urologic: aspiration, injection with phenylephrine	NS	NS
Tazi [57]	2009	33	Y	400	22 h	N	N	N	Urological: aspiration, irrigation, injection with epinephrine	Y, “later”	N

Abbreviations: ED, erectile dysfunction; N, no; NA, not applicable; NS, not specified; WBC, white blood cells; Y, yes.

^a^ Third episode requiring ED‐visit (prior treatment: pseudoephedrine, icepack with result, second: terbutaline without success → aspiration and irrigation).

^b^ Aspiration was performed in other hospital before referral.

^c^ Two episodes in period of 2 months.

^d^ Diagnosed at age of 19 years.

**TABLE 2:** **Overview** of case reports with multiple patients and reviews

First author	Year of publication	
Ali [6]	2021	Systematic review about the characteristics and management options of priapism caused by CML. Sixty‐eight articles, concerning 102 patients with a mean age of 27.4 years. Nearly a quarter of the patients developed permanent ED. Conservative and medical therapy without urological intervention is less likely to be sufficient. Starting treatment of CML to decrease the high WBC count might accelerate the resolution of the priapism and sometimes is needed for a complete resolution.
Chowdhury [58]	2020	Retrospective study to evaluate the need of urologic intervention in priapism in CML patients. Among 12 patients, six were diagnosed as priapism with CML. Mean age was 46.7 years, five patients were newly diagnosed with CML. Of the six patients, five were managed with minimal invasive procedure (injection, aspiration, irrigation), and only one patient needed proximal shunt. After emergency management, all patients were referred to hematologist for definitive management of CML.
Ekeke [59]	2015	Prospective study to evaluate management of priapism in adult men. Sixteen patients had hematological disorders, of which two patients were diagnosed with CML. Precise treatment modalities in these CML patients are not stated.
Jandial [60]	2019	Retrospective study of outcome of priapism in CML patients. Twenty‐three patients (1.7%) of a total of 1350 CML patients had priapism at diagnosis. Median age was 24 years (range 13–50 years). Median duration of priapism was 8 days (range 2–25 days). Baseline median leukocyte count was 285 × 10^9^/L. All patients received cytoreductive therapy (hydroxycarbamide and imatinib), 21 patients underwent penile aspiration, 13 therapeutic leukapheresis, and eight patients distal shunt surgery. ED could be assessed in 14 patients on follow‐up. The occurrence and severity of ED was unaffected by leukapheresis or shunt surgery. Despite favorable response to treatment, long duration of symptoms and hyperleukocytosis probably contributed to ischemic priapism and severe ED in the study cohort.
Kumar and Garg [61]	2018	Review of outcomes of patients with ischemic priapism malignant and non‐malignant. Twenty‐four patients presented with priapism due to CML. Average age at presentation was 27.50 years (SD 6.45), with a mean duration of existing priapism 4.25 days (SD 1.42). No oncological treatment is described. Six patients underwent therapeutic aspiration and irrigation with sympathomimetics, 12 received a distal shunt, and six patients received a proximal shunt. Of these patients, eight had successful detumescence.
Kurosawa [62]	2015	Retrospective cohort concerning 256 children aged less than 20 years, with diagnosis of CML. Four patients experienced priapism. Three of four patients underwent therapeutic aspiration (with or without irrigation) and two of these underwent leukapheresis. Two patients may have been left with ED, despite undergoing treatment with imatinib and hydroxycarbamide, as well as leukapheresis and aspiration or irrigation.
Nabi [63]	2000	Seven patients, aged 8–13 years, time to treatment 2–5 days. Therapy with cavernoso‐spongosal shunts, antileukemic chemotherapy, and radiotherapy. Successful detumescence achieved (mean after 2 days) in all cases with transglandular corpora spongiosum (Winter) shunts and hydroxycarbamide and allopurinol, with one case requiring local radiotherapy. None of patients was potent on follow‐up.
Pal [64]	2015	Prospective, observational study. Nineteen patients presenting with priapism. In one patient, CML was the inducing cause of priapism. No oncological treatment is described, further details of treatment of priapism in this patient is not specifically specified.
Tendulkar [65]	2017	Retrospectively leukapheresis carried out over 4 years, in patients with leukocyte count >100 × 10^9^ and with symptoms of leukostasis. With a mean age of 34 years. Mean initial leukocyte count 312, which reduced to 208 at the completion of all leukapheresis sessions. On average 1.6 sessions were needed.

Abbreviations: CML, chronic myeloid leukemia; ED, erectile dysfunction; SD, standard deviation; WBC, white blood cell.

### 4.3 Overview of evidence concerning different treatment modalities

#### 4.3.1 Case reports

**FIGURE 2:**
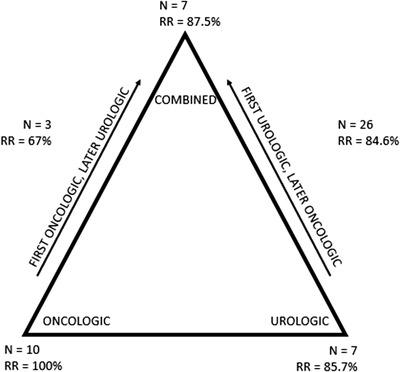
Different treatment modalities (*N*: number of patients). RR: response rate for achieving resolution

The 48 case reports showed a total of 53 cases describing priapism in CML patients (Table 3 and Figure 2). The youngest patient was 9 years old and the oldest patient was 55 years old (mean age 25.3, SD 12.7). Most patients had priapism as a first presentation of CML (*n* = 50, 94.3%). Two patients had a history of CML, one of these patients developed priapism promptly after discontinuation of CML treatment. The mean duration of symptoms before presentation was 2 days, and mean WBC count was 344 × 10^9^/L (SD 147). In most patients, platelet count was in normal range (*n* = 23, 43.4%). Fifteen of the included patients (28%) showed thrombocytosis, ranging from 470 to 1235 × 10^9^/L. Treatment modalities included medications, aspiration, and irrigation to corpora cavernosa, surgical shunts, radiotherapy, and leukapheresis. Most patients received a combination of treatment modalities (*n* = 36, 67.9%). Urological management of priapism by penile aspiration (and irrigation) was used in 41 patients (77.4%), and 14 patients underwent shunt surgery (26.4%). Three patients received penile radiation therapy (5.7%). Oncological management by hyperhydration in combination with hydroxycarbamide was started in 43 patients (81.1%) and chemotherapeutic methods (TKI, chemotherapy) were used in 30 patients (56.6%). In a minority of the patients, leukapheresis was used (*n* = 12, 22.6%). A majority of the patients had resolution of priapism after treatment (*n* = 45, 84.9%). Presence of ED was often not reported, resulting in 60% missing data. Eight (15.1%) patients were reported to experience ED at follow‐up.

**TABLE 3:** Baseline characteristics and outcomes of included patients, totals

Parameters	Value
No. of patients studied	53
Age (year), mean ± SD	25.3 ± 12.7
First presentation, yes	50 (94.3%)
History of CML, yes	2 (3.8%)
Average duration of symptoms (days), median (Q1–Q3) (missing 11%)	2 (0.58–4.0)
WBC count, ×10^9^/L (mean ± SD)	344 ± 147
Platelet count, ×10^9^/L, yes (missing 19%)	
<150	5 (9.4%)
150–450	23 (43.4%)
>450	15 (28%)
Treatment received	
Combined	36 (67.9%)
Unknown timeline/simultaneously	7 (13.2%)
First urological	26 (49.1%)
First oncological	3 (5.7%)
Radiation	3 (5.7%)
Urological	
Penile aspiration ± (alpha adrenergic agonist) irrigation	41 (77.4%)
Shunt (distal/proximal)	14 (26.4%)
Oncological	
Hyperhydration + allopurinol, hydroxycarbamide	43 (81.1%)
Chemotherapeutic methods (chemo, TKI)	30 (56.6%)
Leukapheresis	12 (22.6%)
Resolution, yes (missing 11%)	45 (84.9%)
Erectile dysfunction, yes (missing 60%)	8 (15.1%)

*Note*: Numbers (% of total number patients) unless otherwise specified. Missing data when >10% is mentioned.

Abbreviations: CML, chronic myeloid leukemia; Q1, quartile 1; Q3, quartile 3; SD, standard deviation; TKI, tyrosin kinase inhibitor; WBC, white blood cells.

The case reports were divided by treatment strategy to compare characteristics and outcomes (Table 4). In seven reports, priapism was treated with only oncological treatment modalities [12, 13, 15, 66–18]. This subgroup consisted of 10 patients, with a median age of 18 years, presenting with priapism after a median time of onset of 3.5 days (Q1–Q3 1–10) and a median WBC count of 280 × 10^9^/L. Treatment consisted of hyperhydration and cytoreductive therapy. In five cases, leukapheresis was used [12, 13, 18, 66]. Penile radiation therapy was used in one case report [67]. All patients reached detumescence; however, time to resolution was reported in four cases to cost 5 days–4 weeks. Three patients reported ED, and of the other seven patients this was not reported.

**TABLE 4:** Baseline characteristics and outcomes of included patients, divided by treatment strategies

	Oncologic	First oncologic, later urologic	Combined/unknown timeline	First urologic, later oncologic	Urologic
First author	Atas, Castagnetti (*n* = 3), Ergenc, Kumar (*n* = 2), Musa, Ocheni, Veljkovic	Becerra, Gupta, Morano	Gaye, Hazra, Huei, Misha, Purohit Syarif, Sossa Mel	Abd El Salam, Avci, Chang, Clark, Dhar, Dogra, Ervie, Farhan, Gupta and Agrawal, Jameel (*n* = 2), Khan, Manuel, Minckler, Narendra, Nerli, Rajabto, Patil, Ponniah, Qu, Sachdeva, Shaeer, Singh Dhanjum Swapna, Thakur, Ullah	Almeena, Ammouri, Htun, Ocheni, Sareen, Sun, Tazi
No. of patients studied	10	3	7	26	7
Age (years)	18 (9.0–21.8)	23 (12–NA)	27 (17–46)	20.5 (18–31)	27 (21–36)
WBC count, ×10^9^/L	280 (192–416)	364 (282–NA)	354 ± 163	295 (215–401)	450 (377–501)
Time to treatment (days)	3.5 (1–10)	3 (2–NA)	2 (1–5)	1 (0.38–4.0)	0.92 (0.51–2.21)
Resolution, yes	10 (100%)	2 (67%) NB: 33% missing	6 (87.5%) NB: 14% missing	22 (84.6%) NB: 12% missing	6 (85.7%) NB: 14% missing
Erectile dysfunction, yes	3 (30%) NB: 70% missing	0 (0%) NB: 67% missing	2 (28.6.%) NB: 57% missing	4 (15.4%) NB: 50% missing	0 (14.3%) NB: 86% missing

*Note*: Numbers (% of total number patients) unless otherwise specified. Missing data when >10% is mentioned. Parameters are presented as mean ± standard deviation when normally distributed, or as mean (Quartile 1–Quartile 3) when not normal distributed.

Abbreviations: NA, not applicable; WBC, white blood cells.

Urological treatment alone was chosen in seven case reports [17, 52–57]. Seven patients with median age of 27 years received penile aspiration and some subsequently received penile injection with epinephrine. One patient needed shunt surgery. Six patients achieved detumescence, and in the other patient this was not reported. The presence of ED was only presented for one patient, and he did not experience any disfunction [57].

In a majority of the case reports, a combined treatment was chosen. In three reports, treatment was started with oncological management, consisting of hyperhydration and allopurinol, with the addition of hydroxycarbamide in one case, and use of leukapheresis in another case [20, 21]. In these cases, subsequently urological treatment modalities were introduced, consisting of aspiration and irrigation in all three reports, after which shunt surgery was needed for two patients [19, 21]. Median age was 23 years with a median time to treatment of 3 days. Mean WBC count was 364 × 10^9^/L. Resolution of priapism was reported in two patients, and in the other patient this was not reported. One patient reported no ED; however, data of the other two patients were missing.

In seven reports, a combined approach was chosen, but the specific timeframe of induction of these therapies was not specified in all reports [22, 23, 24, 25, 26, 27, 28]. All used penile aspiration, in five patients subsequently followed by injection with phenylephrine. Four patients underwent shunt surgery [25, 27, 28, 68, 69]. Oncological management consisted of hyperhydration and cytoreductive therapy in all cases, except one [23]. One case reported the use of leukapheresis, in which five sessions were needed [25]. In these reports, median age was 27 years with median WBC count of 354 × 10^9^/L. Time to presentation was relatively short (median 2 days), with only one case presenting after 10 days of complaints [28]. Resolution of priapism was reported in six reports (87.5%), and ED was reported in three cases with two patients suffering from ED.

Of the combined treatment modalities, primary urological treatment and later introducing oncological treatment is most often described [3, 29–41, 43, 45–42, 44, 47, 48] [14, 51]. All patients received penile aspiration except three. Narendra et al. reported that urological consultation was performed, but due to short duration of priapism with mild pain and lack of ischemia conservative management was advised. In Dogra et al. [34] and Gupta et al. [37], shunt surgery was directly performed without penile aspiration as first step. In these cases, time to treatment consisted of, respectively, 2 and 10 days, with WBC in higher ranges, 320 × 10^9^ and 420 × 10^9^/L. A total of seven patients received shunt surgery, five after unsuccessful detumescence after penile aspiration and injection [30, 32, 33, 47, 50]. Two patients were treated with a third modality and received penile radiation therapy [40, 49]. Oncological management consisted of hyperhydration and cytoreductive therapy for all patients. Some case reports reported start of treatment with chemotherapy or TKI when diagnosis of CML was established. Five cases used leukapheresis, the amount of sessions performed is not reported [3, 32, 40, 46, 49]. In 85% of the patients, detumescence was reached after treatment, 15.4% (*n* = 4) reported ED.

A total of 11 reports used leukapheresis in treatment of the hyperviscosity causing priapism, consisting of 12 patients (Table 5). Eight case reports used leukapheresis in a combined treatment modality with urological interventions [25, 30, 32, 40]. Three reports used only an oncological approach [12, 13, 18]. The mean WBC count was 365 × 10^9^/L (SD ±4.7), and median time to treatment was 3 days (Q1–Q3 1–5). Ten patients reached detumescence after treatment, one patient experienced still phallus rigidity and tenderness after treatment, and for one patient this was not reported [3, 32].

**TABLE 5:** Baseline characteristics and outcomes of included patients which received leukapheresis

	Oncologic
First author	Atas, Avci, Castagnetti (*n* = 2), Clark, Mishra, Manuel, Morano, Ponniah, Shaeer, Swapna, Veljkovic
No. of patients studied	12
Age (years)	17.3 (±4.7)
WBC count, ×10^9^/L	365 (±193)
Time to treatment in days	3 (1, 2, 3, 4, 5)
Resolution, yes	10 (83.3%) NB: 8.3% missing
Erectile dysfunction, yes	2 (16.7%) NB: 58% missing

*Note*: Numbers (% of total number patients) unless otherwise specified. Parameters are presented as mean ± standard deviation when normally distributed, or as mean (Quartile 1–Quartile 3) when not normal distributed.

### 4.4 Observational studies and reviews

Additionally, we included five retrospective studies, two prospective observational studies and two reviews concerning treatment of malignant priapism (Table 2). Two studies evaluated urological management in malignant priapism. In Chowdhury et al. [58], the need of urological interventions for priapism in CML patients was evaluated. Five of six patients managed full detumescence with solely minimal invasive urological management (injection, aspiration, irrigation) and just one patient ultimately needed shunt surgery. In Kumar et al. [61], outcomes of urological management resulted in a successful detumescence in eight out of 24 patients (33%, six patients aspiration and irrigation, 18 shunt surgery).

In Kurosawa et al. [62], three of four patients underwent therapeutic aspiration, with two additionally followed by leukapheresis. Despite combination of urological and oncological management, these patients were left with ED. The high incidence rates of ED after experiencing priapism are further demonstrated in a retrospective cohort of Nabi and Dogra [63], consisting of seven patients. Therapy with shunt surgery, antileukemic therapy, and radiotherapy resulted in successful detumescence in all patients (mean duration of 2 days); however, all patients experienced ED at follow‐up.

The outcomes of priapism in CML patients were studied in a retrospective cohort by Jandial et al. [60], 23 patients had priapism at diagnosis. All patients received cytoreductive therapy (hydroxycarbamide and Imatinib), additionally 21 patients underwent aspiration, 13 patients therapeutic leukapheresis, and eight patients shunt surgery. ED could be assessed in 14 patients at follow‐up, occurrence and severity of ED were unaffected by leukapheresis or shunt surgery. In a study by Tendulkar et al. [65], leukapheresis was found to be adequate in lowering leukocyte count, with an average of 1.6 sessions needed. Despite the favorable response in Jandial et al., long duration of symptoms and high leukocyte counts may have contributed to ischemic priapism and severe ED.

In a recently published systematic review about characteristics and management options of priapism caused by CML by Ali et al. [6], 102 patients were evaluated. Nearly, a quarter of these patients developed permanent ED. They state that conservative and medical therapy without urological intervention is less likely to be sufficient; however, treating CML to decrease the WBC may accelerate resolution and is therefore advised.

In two prospective observational studies, management of priapism in CML was evaluated [59, 64]. However, specific treatment modalities were not stated in these reports.

## 5 DISCUSSION

Priapism in CML is an urological emergency and it must be treated promptly to rapidly restore arterial inflow and venous outflow. Treatment aims to relieve symptoms and to prevent ED. When reviewing literature concerning treatment modalities of low flow priapism, three different modalities can be differentiated: radiation therapy, and oncological and urological managements. In this review, we evaluated the existing data after introduction of the TKIs concerning treatment of low flow priapism, and evaluated the value of leukapheresis.

Ischemic priapism is characterized by reduced intracavernous blood flow, and is a type of compartment syndrome leading to stasis, acidosis, and hypoxia. It is predicted if it remains untreated within 24–48 h, it can result in irreversible damage and fibrosis, leading to ED. In CML patients, the hyperleukocytosis is considered to be the cause of priapism. Hyperleukocytosis is defined as WBC >100 × 10^9^/L and it can present clinical symptoms of hyperviscosity such as shortness of breath, central nervous system disorders, impaired kidney function, or priapism [70]. There are little data available on the correct management of priapism, especially leukemic priapism. Due to the rare occurrence and small case series, no standard guideline or treatment is recommended for leukemic priapism [1].

Treatment of CML consists of cytoreductive therapies, such as high dose hydroxycarbamide and TKIs, sometimes with addition of leukapheresis to reduce hyperviscosity. The introduction of TKIs has made CML a functionally curable chronic disease and life expectancy for the vast majority of chronic phase CML patients is “normal.” Patients with CML receiving TKI could be expected to have a survival and quality of life comparable to healthy people of their age and sex [71]. The SOKAL‐score of our patient indicated high risk CML. As described by Ciftciler and Haznedaroglu [71], in patients with higher risk disease second‐generation TKI as first‐line therapy has proven to be more beneficial in terms of early and deeper response rates. In a population‐based cohort by Geelen et al. [72], all response milestones were achieved faster in patients treated upfront with a second‐generation TKI but patients initially treated with Imatinib also reached similar levels of response. Imatinib has become a cost‐effective initial treatment for CML and is globally available, and is therefore first choice of treatment of chronic phase CML in our center. Although, treatment with TKIs is commenced immediately after confirming the diagnosis of CML, most often this is a few days after the patient presents acutely. For immediate lowering of WBC, high dose hydroxycarbamide can be initiated, and this will reduce the WBC count by up to 60% after 24–48 h [73, 74]. To further lower WBC, leukapheresis can be initiated. Leukapheresis can reduce the number of blast cells faster but the procedure alone is associated with rebound of blast cells because the removal of blast cells is not accompanied by destruction of blast cells in bone marrow, which can quickly replace the removed blast cells in peripheral circulation [75, 76, 77]. Leukapheresis can be given in conjunction with chemotherapy such as hydroxycarbamide to establish lowering of WBC as quickly as possible to prevent complications of leukostasis. The value of leukapheresis in addition to chemotherapeutic modalities is yet to be evaluated for CML, hence this paper. Literature concerning AML patients receiving both chemotherapy and leukapheresis did not show better early and long‐term survival rates [70, 78]. However, the disease course of AML and CML are not comparable. AML is a fast moving leukemia where mortality of leukostasis is 40% and occurs usually within the first few weeks of diagnosis, whether CML is most often a slowly progressive disease, which can be considered chronic with the advent of TKIs. Results of leukapheresis in AML may therefore not be applicable to CML.

An expert panel discussion and review of available data‐based guideline of treating leukemic priapism has been published by the American Urological Association, resulting in a number of recommendations [8]. The strongest recommendation is that ischemic priapism requires treatment directed at the penis primarily, by using aspiration (and irrigation) or shunt surgery. In ischemic priapism, “time is erectile tissue.” After 12 h, trabecular edema is seen, after 24 h, platelets adhere to the sinusoids, and after 48 h, necrosis and thrombi are seen. Ninety percent of men with ischemic priapism lasting more than 24 h develop ED [79]. A step‐wise management may be used, starting with penile aspiration which may be used both as a diagnostic and therapeutic measure. Aspiration may be combined with irrigation with normal saline. Aspiration with or without irrigation is successful in 24%–36% of the cases of priapism [6]. If no detumescence is achieved, intracavernous injection of sympathomimetics should be performed. When conservative measures fails, priapism can be treated with surgical methods [2, 6]. However, in malignant priapism, such as in CML, it is less frequently the only therapy of choice and was only chosen in seven case reports. All patients received aspiration and penile injection with epinephrine, only one patient needed shunt surgery, leading to a success rate of 85.7% solely based on these case reports, in contrary to the 30% success rate often reported for aspiration alone [1]. The outcome of ED is not presented in six of these reports.

Most cases chose a combined approach because urologic treatment alone does not tackle the underlying cause of the hyperleukocytosis. The primary mechanism in CML is the aggregation of leukemic cells in the corpora cavernosa and the dorsal veins of the penis, simultaneously venous congestion of corpora cavernosa and infiltration of the sacral nerves may play a role [38]. The oncological approach consists of chemotherapy, radiotherapy, or leukapheresis. We found three case reports using radiation therapy as treatment modality; however, no in‐depth literature concerning this treatment is available.

The evidence of treatment with chemotherapy alone is supported by limited amount of literature. A number of case reports have reported successful use of oncological agents alone, with all patients reaching detumescence. However, time to detumescence was reported to cost 5 days–4 weeks. Two points should be considered; detumescence may be explained by the mechanism of action of the chemotherapeutic managements in which lowering of leukemic cells concentration takes time, or achieving detumescence might be the natural course of priapism rather than a true treatment success [8].

Therapeutic leukapheresis is an oncological treatment modality, which can accelerate the lowering of leukemic cells. We found 11 case series that have reported the successful use of therapeutic leukapheresis to treat priapism. Eight case reports combined this with urological interventions; in the other three reports, it was combined with only oncological management. A meta‐analysis by the American Urological Association found that three of four patients treated by leukapheresis had resolution of priapism compared to only three of 15 patients treated with chemotherapy alone [8]. Leukapheresis can rapidly reduce the number of leukocytes compared to chemotherapy to improve the microcirculation, and is reported to reduce leukocytes count up to 10%– 70% [77]. However, leukapheresis comes with high costs and requires specialized equipment, trained personnel, and need of a central venous catheter, which can cause a delay in treatment. Another disadvantage is the loss of red blood cell and platelets, so its suitability in patients with low Hb or platelets levels is limited [80]. However, we state that the disadvantages do not outweigh the benefits of leukapheresis. Leukapheresis can decrease leukocyte count rapidly in approximately 1.6 sessions, and minimize complications caused by hyperviscosity and leukocyte aggregation in microcirculations [65]. Secondly, leukapheresis could decrease the occurrence of tumor‐lysis syndrome in leukemia patients [81]. In case of hyperviscosity, we recommend to start therapeutic leukapheresis alongside systemic therapy to lower leukocytes as quickly as possible. In waiting time for the leukapheresis, start of chemotherapy should not be postponed.

**FIGURE 3:**
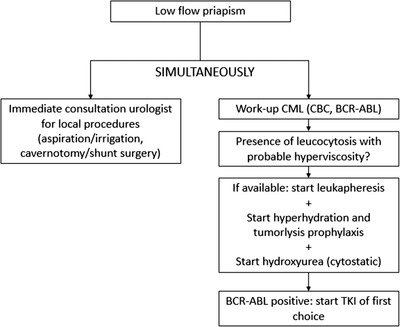
Proposed workflow in low flow priapism

The primary goal of treating priapism in CML is to achieve detumescence as quickly as possible to preserve erectile function. Therefore, we state in accordance with the American Urological Association guideline of treating priapism and a recently published review, that treatment of CML should be a combination of oncological treatment alongside urological treatment, and neither treatment modality should be used as only treatment [8]. When presenting with malignant priapism, immediate intracavernous treatment is required and should be administered in a step‐wise fashion until detumescence is achieved; first therapeutic aspiration, followed by injection of sympathomimetics, and after multiple trials of penile injection, shunt surgery should be considered. Treating the underlying hyperviscosity caused by CML can decrease WBC and further accelerate resolution. When CML is suspected, we advise to start therapeutic leukapheresis when no other contraindications are present. Simultaneously, patients should be started on cytoreductive therapy and this should not be postponed when waiting to start leukapheresis (Figure 3).

### 5.1 Limitations

Some limitations of these recommendation should be noted. Due to the relatively rare condition, literature concerning treatment and outcomes is scarce. This recommendation is therefore solely based on case reports, reviews, and expert opinions. The primary goal of treating priapism, not only in CML patients, is to resolve the erection as soon as possible to prevent ED. Detumescence, time to detumescence, and presence of ED at follow‐up are the main outcomes to evaluate different treatment modalities. However, presence of ED was not reported in 60% of the included case reports, which complicates the interpretation of the results and clinical relevance of these treatments. Furthermore, we only included articles published after 2000 because of the introduction of TKIs as a gold standard of CML treatment. With the use of TKIs, treatment results greatly differ from previously used methods, which could bias results and might not reflect the true value of leukapheresis.

## 6 CONCLUSION

Priapism can be the first manifestation of a previously unknown CML. Priapism is an urological emergency and when untreated can result in ED. The treatment of priapism in CML should be multidisciplinary; we highlight the importance of local urological therapy combined with systemic therapy for CML. Therapeutic leukapheresis should be applied when available and in case of no other contraindications, next to both other treatment modalities to accelerate the lowering of WBC and therefore resolution of priapism.

## CONFLICT OF INTEREST

The authors declare they have no conflicts of interest.

## FUNDING INFORMATION

The authors received no specific funding for this work.

## AUTHOR CONTRIBUTIONS

*Study concept and design*: Marleen van der Velde, Sanne Tiellemans, Heleen de Lil, and Laurens Nieuwenhuizen. *Acquisition of data*: Marleen van der Velde and Sanne Tiellemans. *Analysis/interpretation of data*: Marleen van der Velde, Sanne Tiellemans, Heleen de Lil, and Laurens Nieuwenhuizen. *Drafting of the manuscript*: Marleen van der Velde and Sanne Tiellemans. *Critical revision of the manuscript*: Heleen de Lil and Laurens Nieuwenhuizen. This manuscript has been read and approved by all the authors.

## ETHICS STATEMENT

Ethics approval was not sought because the data used is from previous published studies in which informed consent is obtained by primary investigators.

## PATIENT CONSENT STATEMENT

This work was published with consent of the described patient.

## Supporting information

Supplementary appendix 1: Search method

## Data Availability

Data sharing is not applicable to this article as no new data were created or analyzed in this study.

## References

[1] Rodgers R , Latif Z, Copland M . How I manage priapism in chronic myeloid leukaemia patients. Br J Haematol. 2012;158(2):155–64. 22571386 10.1111/j.1365-2141.2012.09151.x

[2] Faderl S , Talpaz M , Estrov Z , O'Brien S , Kurzrock R , Kantarjian HM . The biology of chronic myeloid leukemia. N Engl J Med. 1999;341(3):164–72. 10403855 10.1056/NEJM199907153410306

[3] Shaeer OK , Shaeer KZ , Abdel Rahman IF , El‐Haddad MS , Selim OM . Priapism as a result of chronic myeloid leukemia: case report, pathology, and review of the literature. J Sex Med. 2015;12(3):827–34. 25630365 10.1111/jsm.12812

[4] Ganguly S , Lakshmaiah KC , Jacob LA , Babu S , Dasappa L , Govind Babu KS . Performance of Sokal and Eutos scores for predicting cytogenetic and molecular response in newly diagnosed chronic myeloid leukemia‐chronic phase patients on imatinib. Indian J Hematol Blood Transfusion. 2017;33(1):82–6. 10.1007/s12288-016-0667-x PMC5280853 28194061

[5] Ouzzani M , Hammady H , Fedorowicz Z , Elmagarmid A . Rayyan—a web and mobile app for systematic reviews. Syst Rev. 2016;5(1):210. 27919275 10.1186/s13643-016-0384-4 PMC5139140

[6] Ali E , Soliman A , De Sanctis V , Nussbaumer D , Yassin M . Priapism in patients with chronic myeloid leukemia (CML): a systematic review. Acta Bio‐Med. 2021;92(3):e2021193. 10.23750/abm.v92i3.10796 PMC8343736 34212918

[7] Marcu D , Iorga L , Mischianu D , Bacalbasa N , Balescu I , Bratu O . Malignant priapism—what do we know about it? In Vivo. 2020;34(5):2225–32. 32871745 10.21873/invivo.12033 PMC7652445

[8] Montague DK , Jarow J , Broderick GA , Dmochowski RR , Heaton JP , Lue TF , et al. American Urological Association guideline on the management of priapism. J Urol. 2003;170(4):1318–24. 14501756 10.1097/01.ju.0000087608.07371.ca

[9] Levey HR , Segal RL , Bivalacqua TJ . Management of priapism: an update for clinicians. Ther Adv Urol. 2014;6(6):230–44. 25435917 10.1177/1756287214542096 PMC4236300

[10] O'Brien SG , Guilhot F , Larson RA , Gathmann I , Baccarani M , Cervantes F , et al. Imatinib compared with interferon and low‐dose cytarabine for newly diagnosed chronic‐phase chronic myeloid leukemia. N Engl J Med. 2003;348(11):994–1004. 12637609 10.1056/NEJMoa022457

[11] Chisick L , Seftel M , Kumar R . Algorithm for initial management of priapism in chronic myeloid leukaemia. Br J Haematol. 2012;159(2):250–1. 22924590 10.1111/bjh.12015

[12] Atas U , Meydanal YE , Iltar U , Ulas T , Salim O , Undar L . Priapism—a rare presentation of chronic myeloid leukaemia. J Clin Diagnostic Res. 2019;13.

[13] Castagnetti M , Sainati L , Giona F , Varotto S , Carli M , Rigamonti W . Conservative management of priapism secondary to leukemia. Pediatr Blood Cancer. 2008;51(3):420–3. 18506758 10.1002/pbc.21628

[14] Dhanju AS , Tyagi P , Dhaliwal SS , Paul S , Singh R , Singh J , et al. Priapism: a rare presentation in chronic myeloid leukemia. J Int J Adv Med. 2019;6(6):3

[15] Kumar P , Rahman K , Kumari S , Singh MK , Gupta R , Nityanand S . Priapism as a rare presentation of chronic myeloid leukemia. J Cancer Res Therap. 2018;14(6):1442–3. 30488874 10.4103/0973-1482.199388

[16] Musa A , Ndakotsu M , Abubakar S , Agwu P . Chronic myeloid leukemia with an initial presentation as ischemic priapism: a case report and review of literature. Arch Int Surg. 2017;7(2):68–72.

[17] Ocheni S , Ibegbulam O , Olusina D , Oyekunle A , Durosinmi M . Chronic myeloid leukaemia presenting as priapism: a report of 2 cases and review of literature. Int J Med Health Dev. 2010;15(2):76–81.

[18] Veljković D , Kuzmanović M , Mićić D , Šerbić‐Nonković O . Leukapheresis in management hyperleucocytosis induced complications in two pediatric patients with chronic myelogenous leukemia. Transf Apher Sci. 2012;46(3):263–7. 10.1016/j.transci.2012.03.012 22480956

[19] Becerra‐Pedraza LC , Jiménez‐Martínez LE , Peña‐Morfin I , Nava‐Esquivel R , Villegas‐Martínez JA . Priapism as the initial sign in hematologic disease: case report and literature review. Int J Surg Case Rep. 2018;43:13–7. 29414500 10.1016/j.ijscr.2017.12.038 PMC5907689

[20] Gupta A , Seth T , Gupta A . Successful use of terbutaline in persistent priapism in a 12‐year‐old boy with chronic myeloid leukemia. Pediatr Hematol Oncol. 2009;26(1):70–3. 19206011 10.1080/08880010802435146

[21] Morano SG , Latagliata R , Carmosino I , Girmenia C , Dal Forno S , Alimena G . Treatment of long‐lasting priapism in chronic myeloid leukemia at onset. Ann Hematol. 2000;79(11):644–5. 11131926 10.1007/s002770000199

[22] Gaye O , Thiam NM , Cassell A , Gueye SM , Sow Y , Fall B , et al. Unusual presentation of priapism associated with acute and chronic myeloid leukemia in two patients: emergency management. Case Rep Urol. 2020;2020:4982432. 32855834 10.1155/2020/4982432 PMC7442991

[23] Hazra SP , Priyadarshi V , Gogoi D , Sharma PK , Pal DK , Chakraborty SC . Pediatric priapism: a rare first manifestation of leukemia. APSP J Case Rep. 2013;4(3):39. 24381835 PMC3863828

[24] Huei TJ , Lip HT , Shamsuddin O . A rare presentation of chronic myeloid leukaemia with priapism treated with corporoglandular shunting. Med J Malaysia. 2018;73(6):420–2. 30647220

[25] Mishra K , Jandial A , Singh V , Radotra BD , Malhotra P . Priapism in chronic myeloid leukemia: meeting at the crossroads and heading in different directions. Indian J Med Paediatr Oncol. 2020;41:418.

[26] Purohit AH , Sarangi S , Kumar D , Bohra GK , Saha S , Pandey H . Is priapism a common presentation of chronic myeloid leukemia in an adolescent patient? Cardiovasc Hematol Disord Drug Targets. 2021;21(2):147–8. 10.2174/1871529X21666210331142330 33797372

[27] Sossa Melo CL , Orozco CA , Peña Castellanos AM , Rueda Perea MA , Porras Bueno CO , Romero Diaz CI , et al. Priapism as the first manifestation in chronic myeloid leukemia: a case report and focused review of literature. Clin Case Rep. 2021;9(11):e04901. 34824846 10.1002/ccr3.4901 PMC8603417

[28] Syarif, KK , Palinrungi MA , Syahrir S , Azis A , Arfan M . Priapism due to chronic myelocytic leukemia. Urol Case Rep. 2021;40:101946. 34840959 10.1016/j.eucr.2021.101946 PMC8607158

[29] Abd El Salam MA , Ibrahim NH , Hassan S . Discontinuation of treatment in a chronic myeloid leukemia patient caused priapism: a case report. J Human Androl. 2019;9(1):21–3.

[30] Avci AE , Kurtulus F , Fazlioglu A , Keskin S , Güçtaş Ö , Cek M . Priapism as an initial presentation of chronic myelogenous leukemia: a case report. UHOD. 2005;15:153–5.

[31] Chang MW , Tang CC , Chang SS . Priapism—a rare presentation in chronic myeloid leukemia: case report and review of the literature. Chang Gung Med J. 2003;26(4):288–92. 12846529

[32] Clark AJ , Hsu P , Darves‐Bornoz A , Tanaka ST , Mason EF , Katzenstein HM . Case 3: priapism in a 13‐year‐old boy. Pediatr Rev. 2018;39(12):617–9. 30504255 10.1542/pir.2017-0051 PMC7282286

[33] Dhar J , Dhar J , Chhabra G , Khandelwal L , Khandelwal L , Batra A , et al. Priapism as a debut presentation of chronic myeloid leukemia. J College Physicians Surg. 2019;29(1):78–80. 10.29271/jcpsp.2019.01.78 30630577

[34] Dogra PN , Kumar P , Goel R , Dash SC . Long duration priapism in blast crisis of chronic myeloid leukemia. J Assoc Physicians India. 2004;52:170. 15656063

[35] Ervie M , Boongaling DC , Rose S , Mortel C , Deala RP . Priapism as a rare presentation of chronic myelogenous leukemia. Philippine J Inter Med. 2015;53:1–5.

[36] Farhan S , Anjum F , Al‐Qahtani F , Al‐Anazi KJ . Chronic myeloid leukemia presenting with priapism. J Leukemia. 2015;3.

[37] Gupta A , Agrawal P , Aggarwal V , Sathi S. Priapism in CML. Indian J Med Paediatr Oncol. 2008;29(3):30–1.

[38] Jameel T,Mehmood K . Priapisman unusual presentation in chronic myeloid leukaemia: case report and review of the literature. Biomédica. 2009;25:197.

[39] Khan A , Shafiq I , Shah MH , Khan S , Shahid G , Arabdin M . Chronic myeloid leukaemia presenting as priapism: a case report from Khyber Pakhtunkhwa. J Pak Med Assoc. 2018;68(6):942–4. 30323364

[40] Manuel MB , Leak A , Carroll SA . Priapism in the oncology setting. Clin J Oncol Nurs. 2007;11(1):23–5. 10.1188/07.CJON.23-25 17441393

[41] Minckler MR , Conser E , Figueroa JJ , Scott AJ , Gaither J , Amini R . The semantics of priapism and the first sign of chronic myeloid leukemia. Case Rep Emerg Med. 2017;2017:2656203. 28638667 10.1155/2017/2656203 PMC5468559

[42] Narendra R , Shankar LJ , Sandeep J , Bardia MR . Priapism in teenager chronic myelogenous leukemia; a rare occurence. Asian J Pharm Health Sci. 2011;1(4).

[43] Nerli RB , Magdum PV , Hiremath SC , Patil AY , Pai SV , Handigund RS , et al. Priapism—a rare presentation in chronic myeloid leukemia: case report. Urol Case Rep. 2016;4:8–10. 26793565 10.1016/j.eucr.2015.08.005 PMC4719907

[44] Rajabto W , Djianzonie JAC , Pratisthita LB , Shatri H . Priapismus as leukostasis manifestation in chronic myeloid leukemia. Acta Med Indones. 2020;52(4):420–2. 33377887

[45] Patil PSK , Katariya P , Gaikwad N . Priapism—a rare presentation in chronic myeloid leukemia. Vidarbha J Inter Med. 2016;21:50–1.

[46] Ponniah A , Brown CT , Taylor P . Priapism secondary to leukemia: effective management with prompt leukapheresis. Int J Urol. 2004;11(9):809–10. 15379953 10.1111/j.1442-2042.2004.00872.x

[47] Qu M , Lu X , Wang L , Liu Z , Sun Y , Gao X . Priapism secondary to chronic myeloid leukemia treated by a surgical cavernosa‐corpus spongiosum shunt: case report. Asian J Urol. 2019;6(4):373–6. 31768325 10.1016/j.ajur.2018.12.004 PMC6872760

[48] Sachdeva P , Kalra M , Thatikonda KB , Aggarwal SK , Sachdeva D , Sachdeva A . Stuttering priapism in a teenage boy: lesson to be learnt. J Pediatr Hematol Oncol. 2021;43(8):e1118–9. 33235144 10.1097/MPH.0000000000001998

[49] Swapna Y, Narmada B . Emergency leukapheresis in chronic myeloid leukemia presenting with priapism. Asian J Pharmaceutical Health Sci. 2017;7:1701–4.

[50] Thakur P , Verma V , Fotedar V , Singh K . Priapism in a pediatric chronic myeloid leukaemia patient: unusual presentation of a rare disease in children. Clin Cancer Invest J. 2019;8(2):76–8.

[51] Wajih Ullah M , Rehman A , Cheeti A , Siddiq W , Latif WA , Prasai K , et al. Priapism and chronic myelogenous leukemia. Int J Adv Res. 2018;6:144–6.

[52] Almaeena W , Azzuz S . Undiagnosed chronic myelogenous leukemia presented by priapism. Int J Acad Sci Res. 2020;4:20–1.

[53] Ammouri Z , Mouhaoui M . Priapism a rare and unusual presentation in chronic myeloid leukemia (a case report). AMMUR. 2019;3:36.

[54] Htun T , Dublin N , Parameswaran M , Razack A , Chua CJJoH , Chronic myeloid leukaemia presenting as priapism “how should we treat these”. J Health Translat Med. 2008;11(1):27–9.

[55] Sareen R , Kapil M , Malpani BK. Priapism: a rare presentation of CML. J Hematol Oncol Forecast. 2018;1:1–3.

[56] Sun HH , Zhang JH , DeWitt‐Foy M , Waldron M , Mukherjee S , Montague DK . Urologic management of priapism secondary to chronic myeloid leukemia. Urology. 2019;125:24–8. 30471371 10.1016/j.urology.2018.11.021

[57] Tazi I . Priapism as the first manifestation of chronic myeloid leukemia. Ann Saudi Med. 2009;29(5):412. 19700904 10.4103/0256-4947.55176 PMC3290050

[58] Chowdhury ZZ , Al‐Asad H , Rahman MH et al. Management of priapism with chronic myeloid leukaemia—a rare presentation and our experiences. Haematol J Bangladesh. 2020;3:39–41.

[59] Ekeke O , Omunakwe H , Nwauche C . Chronic myeloid leukaemia presenting as priapism. Int Surg. 2015;100:552–7. 25785343 10.9738/INTSURG-D-13-00223.1 PMC4370551

[60] Jandial A , Mishra K , Sandal R , Lad D , Prakash G , Khadwal A ea. CML patients presenting with priapism: is there any disparity in outcome? J Clin Oncol. 2019;37(15).

[61] Kumar M , Garg G , Sharma A , Pandey S , Singh M , Sankhwar SN . Comparison of outcomes in malignant vs. non‐malignant ischemic priapism: 12‐year experience from a tertiary center. Turk J Urol. 2019;45(5):340–4. 30817276 10.5152/tud.2019.75044 PMC6739082

[62] Kurosawa H , Tanizawa A , Tono C , Watanabe A , Shima H , Ito M , et al. Leukostasis in children and adolescents with chronic myeloid leukemia: Japanese pediatric leukemia/lymphoma study group. Pediatr Blood Cancer. 2016;63(3):406–11. 26485422 10.1002/pbc.25803

[63] Nabi G , Dogra PN . Chronic myeloid leukaemia presenting as priapism in children: need for multidisciplinary approach. East Afr Med J. 2000;77(10):576. 12862132

[64] Pal DK , Biswal DK , Ghosh B . Outcome and erectile function following treatment of priapism: an institutional experience. Urol Ann. 2016;8(1):46–50. 26834401 10.4103/0974-7796.165717 PMC4719511

[65] Tendulkar AA , Jain PA , Gupta A , Sharma N , Navkudkar A , Patle V . Therapeutic leukocyte reduction for acute and chronic myeloid leukemias: a 4‐year experience from an oncology center in India. Asian J Transfus Sci. 2017;11(2):156–61. 28970685 10.4103/ajts.AJTS_103_16 PMC5613424

[66] Ergenc H , Varım C , Karacaer C , Çekdemir D . Chronic myeloid leukemia presented with priapism: effective management with prompt leukapheresis. Nigerian J Clin Pract. 2015;18(6):828–30. 10.4103/1119-3077.163282 26289527

[67] Kumar P , Rahman K , Kumari S , Singh M , Gupta R , Nityanand S . Priapism as a rare presentation of chronic myeloid leukemia. J Cancer Res Therap. 2018;14(6):1442–3. 10.4103/0973-1482.199388 30488874

[68] Hazra S , Priyadarshi V , Gogoi D , Sharma P , Pal D , Chakraborty S . Pediatric priapism: a rare first manifestation of leukemia. APSP J Case Rep. 2013;4(3):39. PMC3863828 24381835

[69] Huei T , Lip H , Shamsuddin O . A rare presentation of chronic myeloid leukaemia with priapism treated with corporoglandular shunting. Med J Malaysia. 2018;73(6):420–2. 30647220

[70] Rinaldi I , Sari RM , Tedhy VU , Winston K . Leukapheresis does not improve early survival outcome of acute myeloid leukemia with leukostasis patients—a dual‐center retrospective cohort study. J Blood Med. 2021;12:623–33. 34290537 10.2147/JBM.S312140 PMC8286962

[71] Ciftciler R , Haznedaroglu IC . Tailored tyrosine kinase inhibitor (TKI) treatment of chronic myeloid leukemia (CML) based on current evidence. Eur Rev Med Pharmacol Sci. 2021;25(24):7787–98. 34982440 10.26355/eurrev_202112_27625

[72] Geelen IGP , Thielen N , Janssen J , Hoogendoorn M , Roosma TJA , Willemsen SP , et al. Treatment outcome in a population‐based, ‘real‐world’ cohort of patients with chronic myeloid leukemia. Haematologica. 2017;102(11):1842–9. 28860339 10.3324/haematol.2017.174953 PMC5664388

[73] Adams BD , Baker R , Lopez JA , Spencer S . Myeloproliferative disorders and the hyperviscosity syndrome. Emerg Med Clin North Am. 2009;27(3):459–76. 19646648 10.1016/j.emc.2009.04.001

[74] Porcu P , Cripe LD , Ng EW , Bhatia S , Danielson CM , Orazi A , et al. Hyperleukocytic leukemias and leukostasis: a review of pathophysiology, clinical presentation and management. Leuk Lymphoma. 2000;39(1–2):1–18. 10975379 10.3109/10428190009053534

[75] Tan D , Hwang W , Goh YT . Therapeutic leukapheresis in hyperleukocytic leukaemias—the experience of a tertiary institution in Singapore. Ann Acad Med. 2005;34(3):229–34. 15902342

[76] Singh N , Singh Lubana S , Dabrowski L , Sidhu G . Leukostasis in chronic lymphocytic leukemia. Am J Case Rep. 2020;21:e924798. 32616708 10.12659/AJCR.924798 PMC7360358

[77] Hölig K , Moog R . Leukocyte depletion by therapeutic leukocytapheresis in patients with leukemia. Transf Med Hemother. 2012;39(4):241–5. 10.1159/000341805 PMC3434324 22969693

[78] Malkan UY , Ozcebe OI . Leukapheresis do not improve early death rates in acute myeloid leukemia patients with hyperleukocytosis. Transfus Apher Sci. 2017;56(6):880–2. 29153308 10.1016/j.transci.2017.11.002

[79] Pryor J , Akkus E , Alter G , Jordan G , Lebret T , Levine L , et al. Priapism. J Sexual Med. 2004;1(1):116–20. 10.1111/j.1743-6109.2004.10117.x 16422992

[80] Zhang D , Zhu Y , Jin Y , Kaweme NM , Dong Y . Leukapheresis and hyperleukocytosis, past and future. Int J Gen Med. 2021;14:3457–67. 34285568 10.2147/IJGM.S321787 PMC8286901

[81] Oberoi S , Lehrnbecher T , Phillips B , Hitzler J , Ethier MC , Beyene J , et al. Leukapheresis and low‐dose chemotherapy do not reduce early mortality in acute myeloid leukemia hyperleukocytosis: a systematic review and meta‐analysis. Leuk Res. 2014;38(4):460–8. 24472688 10.1016/j.leukres.2014.01.004

