# Supplementary material for: The value of leukapheresis for treatment of priapism as presenting feature of chronic myeloid leukemia—Case report and review of literature
Source: EJHaem. 2022 Aug 27;3(4):1100–15. doi: 10.1002/jha2.545 (PMC9713041; doi:10.1002/jha2.545)
Supplement: Supplementary file 1 — Supplementary appendix 1: Search method [file JHA2-3-1100-s001.docx]

**Supplementary appendix 1: Search method**

| **Search** | **Query** |
| --- | --- |
| #3 | Search: #1 AND #2 |
| #2 | Search: ("Priapism"[MeSH Terms] OR "priapism*"[Title/Abstract]) |
| #1 | "leukemia, myelogenous, chronic, bcr-abl positive"[MeSH Terms] OR "chronic myelogenous leukemia*"[Title/Abstract] OR "CML"[Title/Abstract] OR "chronic myeloid leukemia*"[Title/Abstract] OR "chronic myelocytic leukemia*"[Title/Abstract]) OR "philadelphia positive myeloid leukemia*"[Title/Abstract]) OR "bcr abl positive"[Title/Abstract]) |
